# Vitamin K epoxide reductase regulation of androgen receptor activity

**DOI:** 10.18632/oncotarget.14639

**Published:** 2017-01-13

**Authors:** Ben Yi Tew, Teresa B. Hong, Maya Otto-Duessel, Catherine Elix, Egbert Castro, Miaoling He, Xiwei Wu, Sumanta K. Pal, Markus Kalkum, Jeremy O. Jones

**Affiliations:** ^1^ Department of Cancer Biology, Beckman Research Institute, City of Hope National Medical Center, Duarte, CA, USA; ^2^ Department of Molecular Immunology, Beckman Research Institute, City of Hope National Medical Center, Duarte, CA, USA; ^3^ Department of Molecular and Cellular Biology, Beckman Research Institute, City of Hope National Medical Center, Duarte, CA, USA; ^4^ Department of Medical Oncology, Beckman Research Institute, City of Hope National Medical Center, Duarte, CA, USA

**Keywords:** prostate cancer, vitamin K epoxide reductase, warfarin, androgen receptor, PPAR

## Abstract

Long-term use of warfarin has been shown to be associated with a reduced risk of prostate cancer. Warfarin belongs to the vitamin K antagonist class of anticoagulants, which inhibit vitamin K epoxide reductase (VKOR). The vitamin K cycle is primarily known for its role in γ-carboxylation, a rare post-translational modification important in blood coagulation. Here we show that warfarin inhibits the transcriptional activity of the androgen receptor (AR), an important driver of prostate cancer development and progression. Warfarin treatment or knockdown of its target VKOR inhibits the activity of AR both in cell lines and in mouse prostate tissue. We demonstrate that AR can be γ-carboxylated, and mapped the γ-carboxylation to glutamate residue 2 (E2) using mass spectrometry. However, mutation of E2 and other glutamates on AR failed to suppress the effects of warfarin on AR suggesting that inhibition of AR is γ-carboxylation independent. To identify pathways upstream of AR signaling that are affected by warfarin, we performed RNA-seq on prostates of warfarin-treated mice. We found that warfarin inhibited peroxisome proliferator-activated receptor gamma (PPARγ) signaling, which in turn, inhibited AR signaling. Although warfarin is unfit for use as a chemopreventative due to its anticoagulatory effects, our data suggest that its ability to reduce prostate cancer risk is independent of its anticoagulation properties. Furthermore, our data show that warfarin inhibits PPARγ and AR signaling, which suggests that inhibition of these pathways could be used to reduce the risk of developing prostate cancer.

## INTRODUCTION

Prostate cancer is the most frequently diagnosed cancer and the second leading cause of cancer mortality in men living in the developed world [1]. The US is expected to spend over $8 billion a year on prostate cancer treatment [2]. The health and financial burdens associated with the screening and treatment of prostate cancer makes it important to identify chemopreventive strategies. The most successful prostate cancer prevention strategies to date have focused on inhibition of the androgen receptor (AR) via blockade of dihydrotestosterone (DHT) production using 5α-reductase inhibitors.

Two large scale studies examined the chemopreventive potential of finasteride and dutasteride, both of which inhibit 5α-reductases, the enzymes that convert testosterone to the more potent androgen DHT in prostate tissue. The prostate cancer prevention trial (PCPT) demonstrated that finasteride reduced the overall risk of prostate cancer in low risk men (relative risk [RR] 0.70, 95% confidence interval [CI] 0.65–0.75) [3]. There has been some concern, however, that finasteride increased the risk of high grade tumors (RR 1.27, 95% CI 1.07–1.50), although the higher incidence of high grade tumors appears to have been due to sampling bias from the effects of the drug on the prostate volume [4]. The reduction by dutasteride of prostate cancer events (REDUCE) trial showed a similar chemopreventive effect with dutasteride (RR 0.77, 95% CI 0.70–0.85) [5]. However, there was no risk reduction of high grade tumors of Gleason score 7 or above. The concern over potential increased risk of high grade tumors and sexual side-effects caused by the drugs has prevented wide-spread adoption of either agent in the chemopreventative setting. Despite this setback, these trials did demonstrate the potential utility of a prostate cancer chemopreventative and the feasibility of testing an agent in this setting.

Multiple retrospective studies have shown that long-term use of warfarin prior to diagnosis is strongly associated with a reduced incidence of prostate cancer. One study examined the use of warfarin in the 5 years preceding diagnosis of urological cancers and discovered that at least 2 years of warfarin use reduced the risk of prostate cancer (RR 0.80, 95% CI 0.66–0.96), but not other urological cancers [6]. The follow-up analysis revealed that warfarin users had lower risk of both low grade and high grade cancers and a lower risk of a poor prognosis based on Gleason score (RR 0.40, 95% CI 0.19–0.83) [7]. Two others studies confirmed these findings, demonstrating that warfarin users had a reduced risk of prostate cancer (RR 0.69, 95% CI 0.50–0.97 and RR 0.86, 95% CI 0.78–0.95), but no reduction in risk of other cancer types [8, 9]. These studies, while necessarily retrospective in nature, convincingly demonstrate that warfarin has specific prostate cancer chemopreventative qualities, in line with the risk reduction observed with 5α-reductase inhibitors, but with additional benefit of reducing high grade cancers.

**Figure 1:**
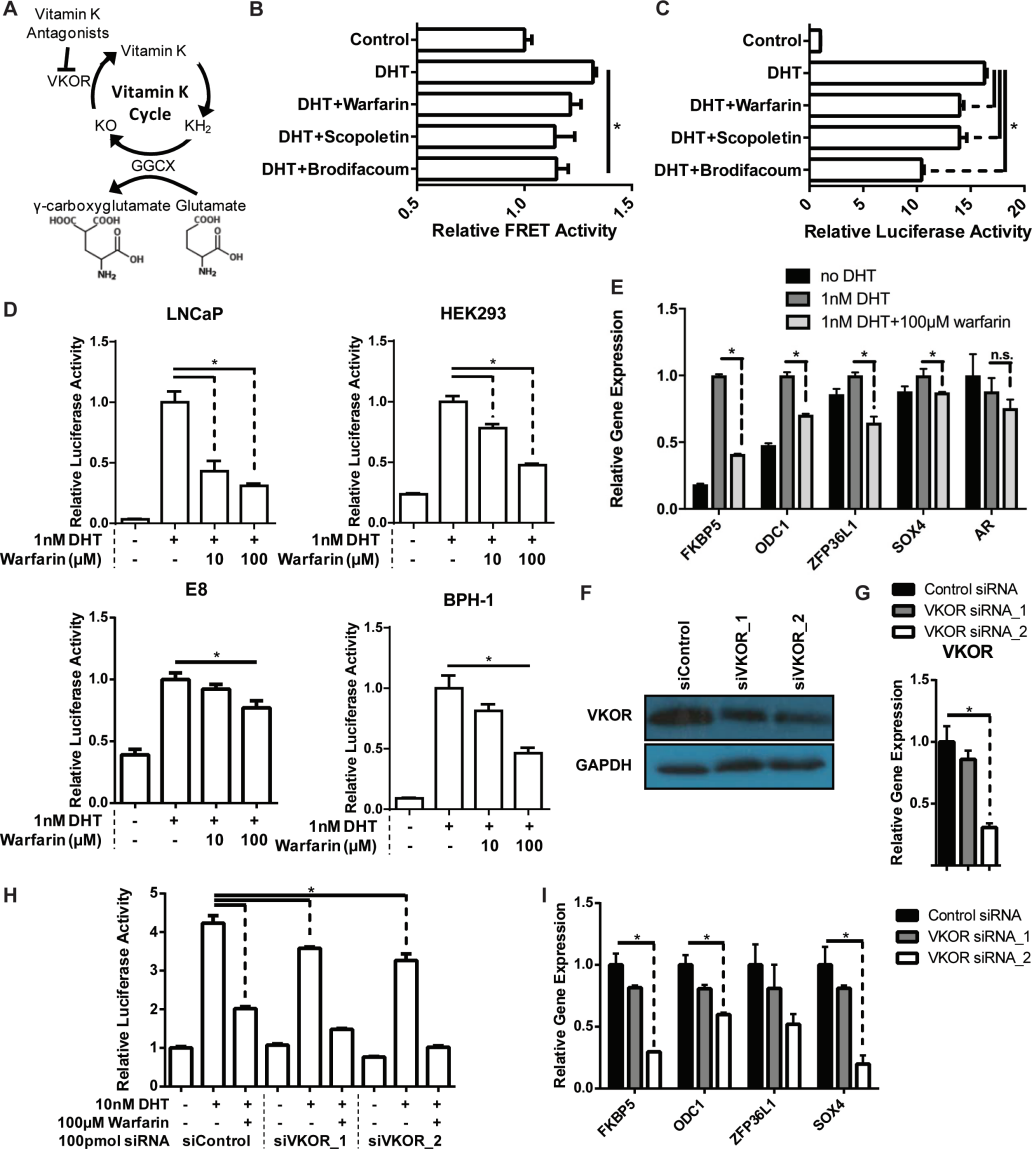
Inhibition of VKOR reduces AR activity (**A**) Schematic of the vitamin K cycle. (**B–D**) The AR conformation change FRET reporter assay (B), luciferase reporter assay (C), and qPCR were used to quantify the effect of vitamin K antagonists on AR activity. (B) HEK293 cells stably expressing the CFP-AR-YFP (CARY) reporter construct were treated with the indicated drugs over night in quadruplicate and the FRET:donor ratio was calculated. (C) HEK293 CARY cells were transfected with luciferase reporters and the following day were treated with the indicated drugs. The following day, transcriptional activity was calculated by normalizing the luciferase activity of the AR-responsive PSA-firefly luciferase reporter to SV40-driven control renilla luciferase reporter activity. Each compound inhibited the DHT-induced AR conformation change and transcriptional activity of AR. (D) Human LNCaP, HEK293 CARY, BPH-1, and mouse E8 prostate cancer cells were transfected with a PSA-luciferase reporter or MMTV-luciferase reporter (E8) as well as the control renilla reporter. The following day, the cells were treated with the indicated drugs and 24 hours later, luciferase activity was quantified. Warfarin inhibits AR activity in a dose dependent fashion in these cells. (**E**) LNCaP cells were treated with the indicated drugs overnight. The following day, RNA was extracted and prepared for qPCR analysis of known AR target genes. Transcript expression was normalized to that of a housekeeping gene. Warfarin reduced the levels of the AR target genes. (**F–I**) VKOR, the target of vitamin K antagonists, was knocked down in HEK293 CARY cells using two different siRNAs. The efficiency of the knockdown was measured by Western blot (F) and qPCR (G). (H) Cells were transfected with siRNAs along with luciferase reporters. The following day, cells were treated with the indicated drugs. VKOR knockdown reduces AR transcriptional activity measured by the luciferase reporter assay. (I) Cells were transfected with siRNAs and the following day were treated with the indicated drugs. The following day, RNA was extracted and prepared for qPCR analysis of known AR target genes. Transcript expression was normalized to that of a housekeeping gene. Both siRNAs against VKOR reduced the levels of the AR target genes.

Warfarin is an anticoagulant that belongs to the class of vitamin K antagonists, which inhibit vitamin K epoxide reductase (VKOR), a key enzyme in the vitamin K cycle (Figure 1A) [10]. Vitamin K participates as a cofactor in γ-carboxylation, a rare post-translational modification where a carboxyl group is added to the γ-carbon of a glutamate residue. Vitamin K is oxidized in this process, and has to be reduced by VKOR before it can participate in the reaction again. By inhibiting VKOR, warfarin prevents the γ-carboxylation of proteins. Currently, only 17 γ-carboxylated proteins have been identified, many of which are involved in blood coagulation [11]. Warfarin is a potent anticoagulant due to the fact that it can prevent the γ-carboxylation of these coagulation factors [12]. However, γ-carboxylated proteins also play other roles, such as in bone formation with the bone gamma-carboxyglutamate protein and periostin [13, 14] and in signal transduction with the Gas6 protein [15]. In addition to γ-carboxylation, vitamin K is also known to be important in other processes such as anti-oxidation and lipid synthesis [16, 17].

The mechanism underlying the association between warfarin use and the reduced risk of prostate cancer is unclear. Because of its role in anticoagulation and the risks associated with that activity, warfarin is not a suitable candidate as a chemopreventive agent. Therefore, it is important to understand the mechanism of action of warfarin and determine if it is possible to separate its chemopreventive properties from its anticoagulation effects. Previously, we identified warfarin and other vitamin K antagonists as AR inhibitors in a high-throughput screen for novel AR regulators [18]. Because inhibition of AR, as seen in the 5α-reductase studies, can reduce the risk of prostate cancer, we hypothesized that the reduced risk of prostate cancer associated with warfarin usage could be mediated, at least in part, by its inhibitory effects on AR signaling. In this study, we sought to understand the mechanism by which warfarin affects AR signaling.

## RESULTS

### Vitamin K antagonists inhibit AR activity by inhibiting VKOR

Warfarin, scopoletin and other coumarins were identified as AR antagonists in a high-throughput screen in HEK 293 kidney cells expressing AR [18]. In addition to these previously identified coumarins, we also tested the ability of brodifacoum [19], a second generation coumarin to inhibit AR activity. All of the vitamin K antagonists tested inhibited both DHT-induced AR conformational change in a FRET reporter assay (Figure 1B) and AR transcriptional activity in a luciferase reporter assay (Figure 1C) in HEK293 cells. The ability of warfarin to inhibit AR transcriptional activity was not limited to HEK293 cells as it also inhibited AR activity in a dose-dependent fashion in LNCaP cells, which are derived from human prostate cancer metastases, BPH-1 cells, which are immortalized benign human prostate epithelial cells [20], and E8 cells, which are derived from localized prostate tumors from Pten knock-out mice [21] (Figure 1D). Warfarin treatment also inhibited the expression of previously identified AR target genes [22], but not AR itself, as determined by RT-qPCR (Figure 1E, Supplementary Figure 1). AR antagonists are known to inhibit the growth of androgen dependent prostate cancer cells. As warfarin has anti-AR activity, we tested its ability to inhibit the growth of LNCaP cells and found that, like the competitive antagonist bicalutamide, warfarin did indeed inhibit the growth of LNCaP cells (Supplementary Figure 2).

Warfarin and other vitamin K antagonists are known to bind and inhibit the VKOR protein. Therefore, we performed siRNA knockdown of VKOR using two different siRNAs and determined the effect on AR activity using a luciferase reporter assay and qPCR of AR target genes. Although knockdown was not complete (Figure 1F, 1G), the siRNAs inhibited AR activity in both assays similarly to small molecule inhibition, suggesting that inhibition of AR by the VKAs is VKOR-dependent (Figure 1H, 1I, Supplementary Figure 3). Warfarin was able to further inhibit AR activity in VKOR depleted cells, likely due to the incomplete knockdown of VKOR.

### Warfarin inhibits AR activity in the mouse prostate

**Figure 2:**
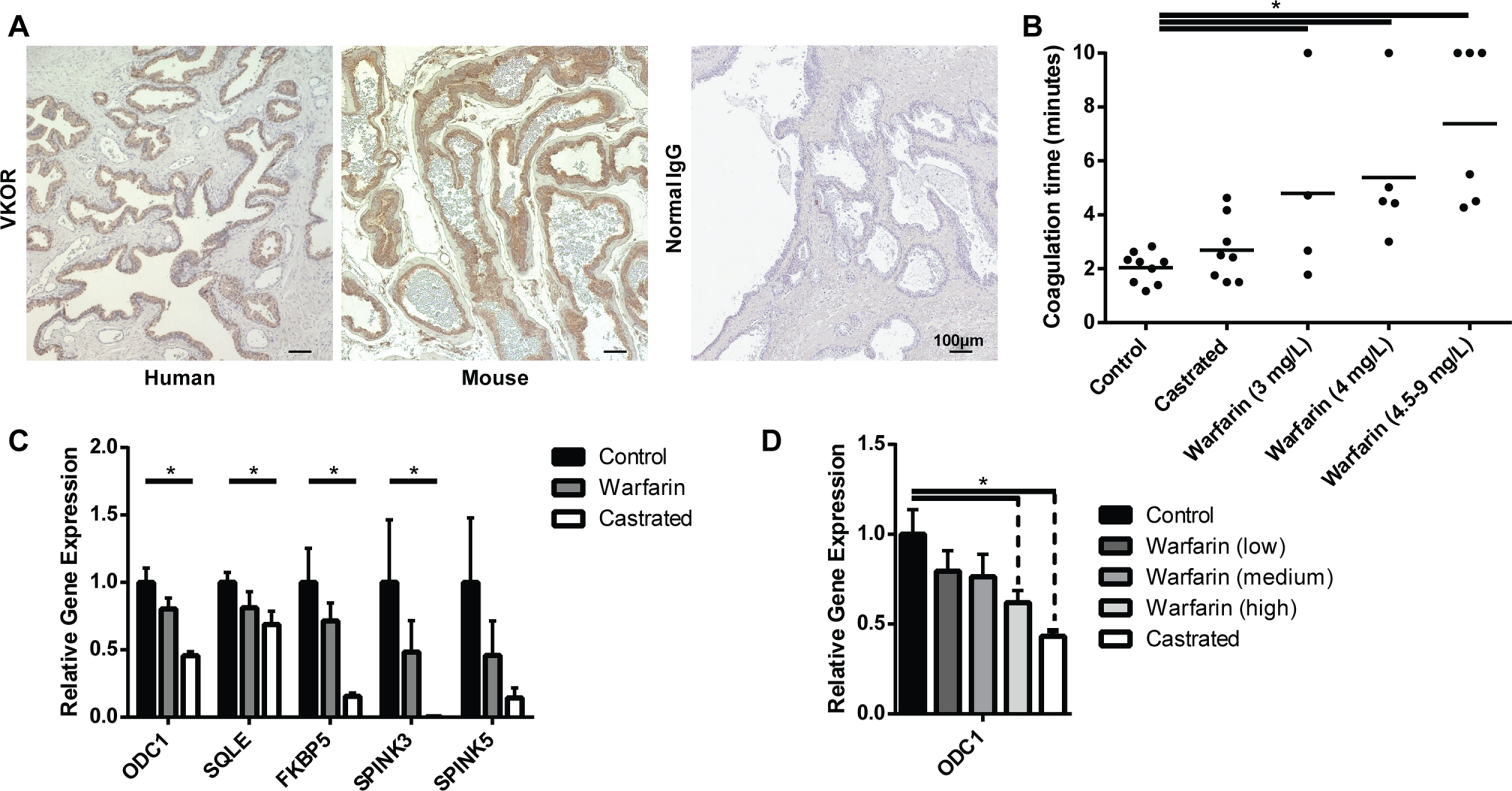
Warfarin treatment inhibits the expression of AR target genes *in vivo* (**A**) Expression of VKOR determined by immunohistochemistry in normal human and mouse prostate. Normal rabbit IgG was used as negative control. VKOR is highly expressed in the prostate epithelia. (**B–D**) Nude mice were treated with high (4.5 mg/L, *n* = 4), medium (4 mg/L, *n* = 5) or low (3 mg/L, *n* = 6) doses of warfarin in their drinking water, castrated (*n* = 8), or left intact (*n* = 9) as controls. After 4 weeks, mice were euthanized and (B) coagulation time was measured. The prostates were also harvested for RNA. The transcript levels of AR target genes were measured by RT-qPCR, with all groups of warfarin treated mice grouped together (C), or separately (D). Differences between control and warfarin treated animals were not found to be significant until broken down by dose for individual genes, such as ODC1.

As a chemopreventative, warfarin likely exerts its effect on benign prostate cells to prevent oncogenic transformation. While it is difficult to test the effect of warfarin treatment in benign human tissue, we can model the effects in mice. Importantly, the target of warfarin, VKOR, is expressed in benign mouse epithelial cells just as it is in benign human epithelial prostate cells (Figure 2A). To test if warfarin inhibited AR target genes *in vivo* in mouse prostate cells as it does in cultured cells, we treated mice for 4 weeks with sub-lethal concentrations of warfarin, as warfarin is a known rodenticide. Mice were also castrated as a positive control for inhibition of AR target genes. The effect of warfarin was confirmed by measuring the clotting time of blood in treated animals (Figure 2B). After four weeks, mice were euthanized and prostate RNA was harvested for quantification by RT-qPCR. Warfarin decreased the expression of AR target genes (Figure 2C). This inhibition, while not as strong as castration, exhibited a dose-dependent response to warfarin at several target genes (Figure 2D, Supplementary Figure 4). Although statistical significance was not obtained at any gene when all warfarin-treated mice were grouped together, several genes were significantly down-regulated when comparing only the high dose of wafarin to control animals, despite having smaller numbers of animals in the warfarin treated group, suggesting a real, reproducible, and biologically relevant response.

### Warfarin inhibits the γ-carboxylation of AR at E2

**Figure 3:**
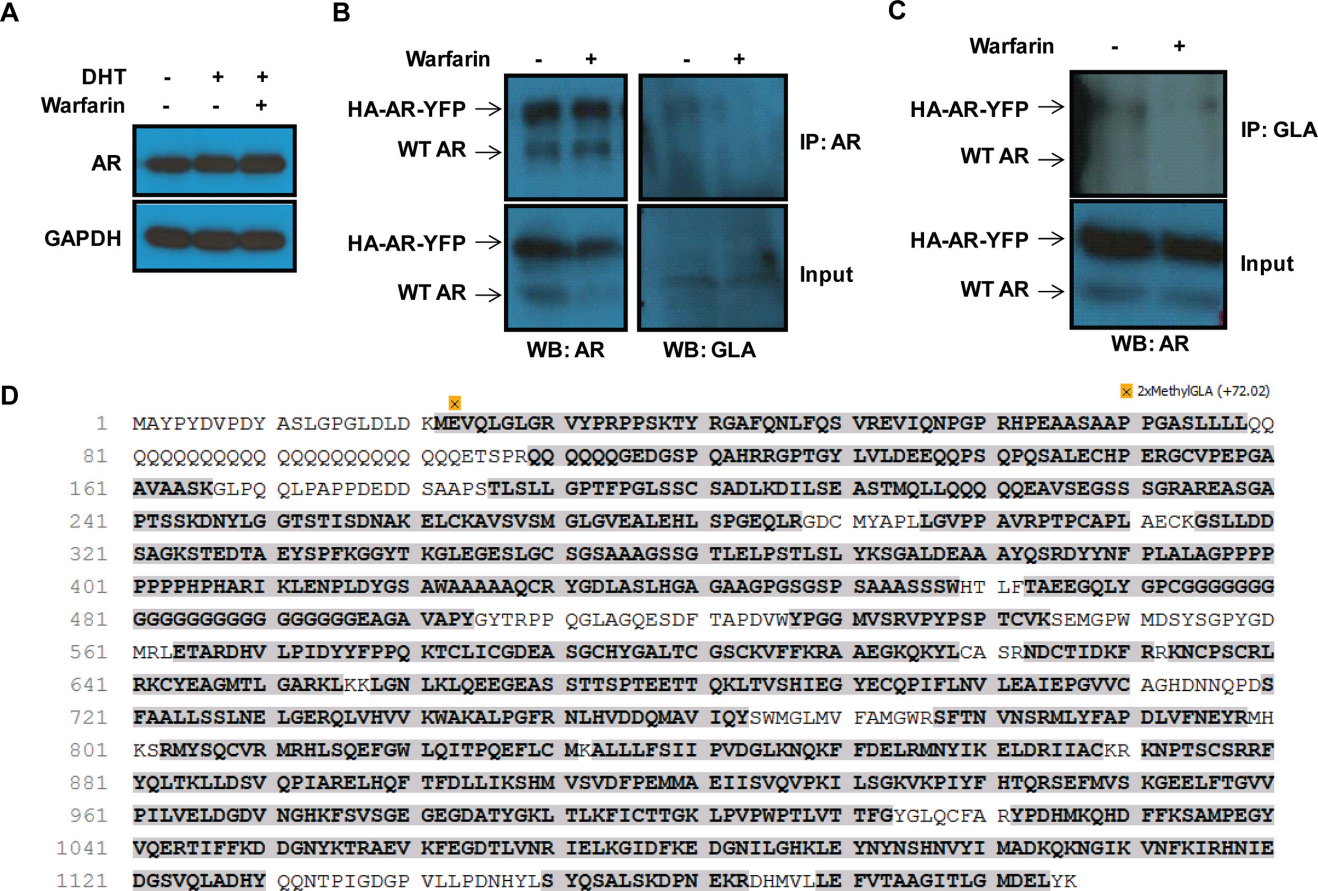
AR is γ-carboxylated at residue E2 (**A**) Expression of AR was determined in LNCaP cells treated overnight with 1 nM DHT and warfarin 100 μM. No change in AR levels were observed after warfarin treatment. (**B**) AR immunoprecipitated from lysate of LNCaP cells stably expressing tagged AR was blotting with γ-carboxyglutamate (Gla) antibody. Gla residues were found on AR, but were no longer present after warfarin treatment. (**C**) Reverse immunoprecipitation from A showed similar results. (**D**) Sequence coverage of AR. 71% coverage of AR and 75% coverage of glutamate residues. HA tag ends at residue 21 while YFP tag begins at residue 944. Residues that were both methylated and γ-carboxylated (2xMethylGLA) with a statistically significant score were labeled.

Since warfarin did not alter the expression of AR (Figure 3A), it is possible that warfarin regulated the activity of AR post-translationally. The primary function of the vitamin K cycle is to generate reduced vitamin K hydroquinone to serve as a cofactor for GGCX, which adds a carboxyl group to the γ-carbon of glutamate to form γ-carboxyglutamate. We therefore hypothesized that AR could be directly γ-carboxylated, and that warfarin could be inhibiting AR activity by preventing its γ-carboxylation. To determine if AR could be modified by γ-carboxylation, we performed AR immunoprecipitation (IP) in LNCaP cells expressing an HA and YFP-tagged AR and blotted with an antibody against γ-carboxyglutamate (anti-Gla) [23]. Both tagged and wild type AR proteins were efficiently immunoprecipitated, and probing with the anti-Gla antibody showed an enrichment at bands corresponding to the tagged and wild type AR (Figure 3B). Importantly, treatment of cells with warfarin prior to IP reduced the detection by the anti-Gla antibody. The reverse experiment, where cell lysates were immunoprecipitated with the anti-Gla antibody followed by blotting for AR, confirmed the presence of γ-carboxylated AR, which was again warfarin dependent (Figure 3C).

We then sought to identify the site of γ-carboxylation on AR using a previously described mass spectrometry method for identifying γ-carboxylglutamate residues on target proteins [11]. Because the γ-carboxylic acid is not stable during the processing of protein samples for mass spectrometry, it must be first protected by methyl ester formation. Thus, we immunoprecipitated AR from control and warfarin-treated LNCaP cells as above, resolved cell lysates on SDS-PAGE, and as part of the digestion and preparation of peptides for analysis, we included a methyl esterfication step (see methods). To identify γ -carboxylated residues we adapted the analysis algorithms to search for glutamate residues containing both methylated and γ-carboxylated glutamates. Using this methodology we attained 71% coverage of AR and 75% coverage of the glutamates (Supplementary Figure 5). Out of the observed glutamates, only glutamate residue 2 (E2) was found to be γ-carboxylated (Figure 3). No other peptide had any γ-carboxylated glutamates that achieved a statistically significant score. Interestingly, a mutation of E2 to K has been found to be responsible for partial androgen insensitivity syndrome (PAIS) [24]. Such a mutation would be expected to prevent γ-carboxylation.

### Warfarin inhibition of AR is independent of its γ-carboxylation

**Figure 4:**
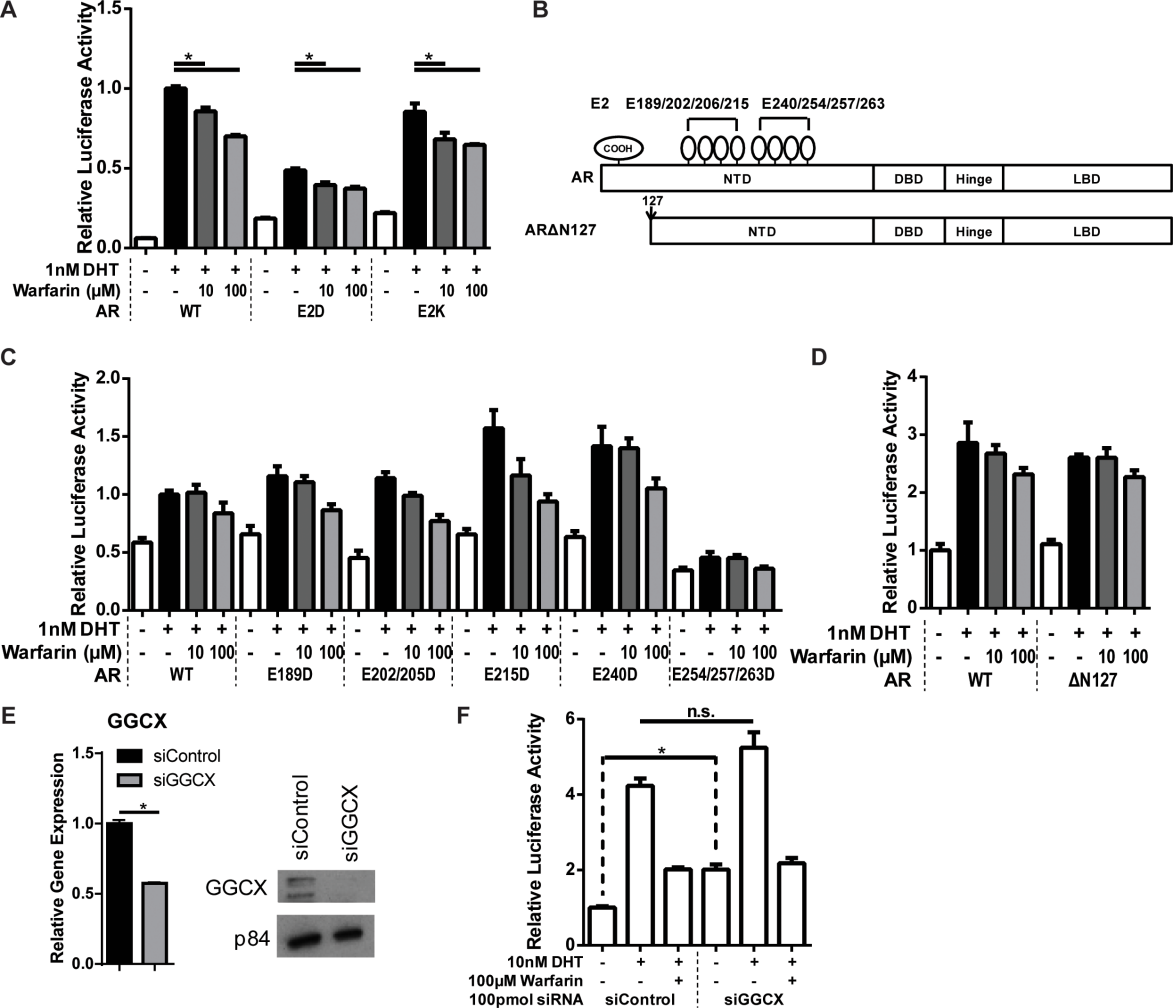
Effect of warfarin on AR transcriptional activity is independent of its γ-carboxylation (**A**) HEK293 cells were transfected with wild type, E2D or E2K mutant AR and assayed for AR activity by PSA-luciferase as previously described. AR mutants remained sensitive to warfarin-mediated inhibition. (**B**) Schematic of the location of probable γ-carboxlyation sites (top) that were mutated by site-directed mutagenesis to either aspartate (E2D) or lysine (E2K) to prevent γ-carboxylation. The N-terminus of AR was also deleted before residue 127 (ΔN127) to remove possible γ-carboxylation cluster. (**C–D**) HEK293 cells transfected with various glutamate mutants of AR as in (A) remained sensitive to warfarin. (**E–F**) GGCX, the enzyme that catalyzes the γ-carboxylation of proteins, was knocked down in HEK293 cells. (E) The efficiency of the knockdown was measured by RT-qPCR. (F) GGCX knockdown increased AR activity, which was opposite of the effect of VKOR.

To determine if E2 γ-carboxylation mediates the effect of warfarin on AR activity, we mutated the E2 residue into aspartate, which would preserve the charge but prevent γ-carboxylation, or lysine, the mutation observed in PAIS patients. Mutation of E2 decreased AR activity in the PSA-luciferase activity, but interestingly, it did not abrogate the inhibitory effects of warfarin (Figure 4A). Mutation of E2 is also known to decrease the binding of the AR co-activator ART-27 to AR and reduce the co-activating effect [24]. While we found that mutation of E2 prevented co-activation in response to ART-27 as expected, warfarin did not prevent the co-activation in response to ART-27 (Supplementary Figure 6A). Furthermore, ART-27 binding to AR was not inhibited by treatment with warfarin (Supplementary Figure 6B). These data combined suggest that, while E2 is important for AR activity, the γ-carboxylation of E2 alone does not mediate the response to warfarin. This suggests two possibilities; there are other γ-carboxylation sites on AR that mediate the response to warfarin or that warfarin is inhibiting AR via a means other than direct AR γ-carboxylation. To test the first possibility, we mutated a series of glutamates that were not covered by our mass spectrometric analysis. Most γ-carboxylated proteins have multiple γ-carboxylation sites in close proximity to each other [25]. We identified several likely clusters in the N-terminal domain of AR (Figure 4B). We mutated these sites and utilized an N-terminal deletion construct of AR; however, none of the glutamate mutations or deletions affected warfarin sensitivity (Figure 4C, 4D). We next performed siRNA knockdown of GGCX itself, the most proximal enzyme in the γ-carboxylation pathway (Figure 4E). Interestingly, GGCX knock down did not inhibit AR activity like VKOR knock down or treatment with VKAs (Figure 4F). This strongly suggests that the AR inhibitory effects of warfarin are not driven by the inhibition of AR γ-carboxylation.

### Warfarin affects multiple pathways in benign prostate tissue

**Figure 5:**
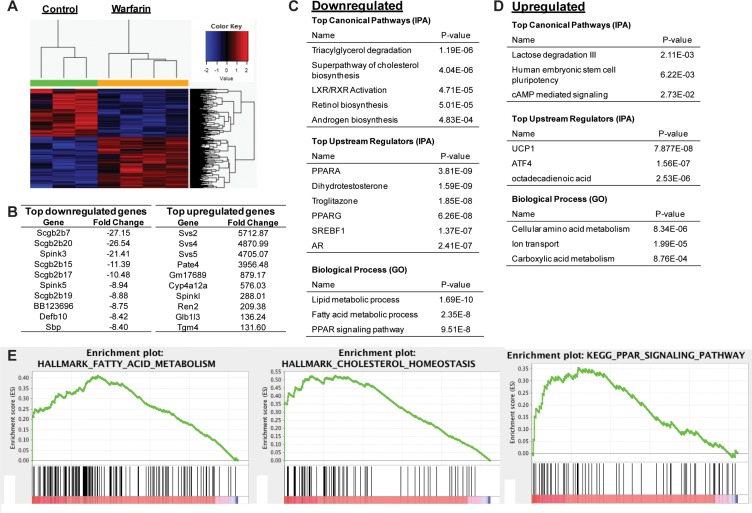
Warfarin inhibits lipid biosynthetic pathways and their upstream regulators RNA-seq was performed on prostate of mice treated with warfarin or left untreated as controls. (**A**) Heatmap of selected genes whose expression significantly changed between warfarin and control groups. (**B**) Top upregulated and downregulated genes identified. (**C, D**) Top (C) downregulated and (D), upregulated pathways identified by ingenuity pathway analysis (IPA) and David functional annotation (GO). (**E**) GSEA analysis demonstrates that fatty acid and cholesterol synthesis pathways are significantly inhibited by warfarin treatment, along with PPAR signaling.

Vitamin K is known to regulate cellular processes other than γ-carboxylation, such as electron transport, anti-oxidation and lipid biosynthesis, which could potentially be inhibited by warfarin [16, 17, 26]. We therefore sought to identify potential pathways that could mediate the effects of warfarin, not just on AR activity, but on chemoprevention of prostate cancer. To do so, we performed RNA-seq on prostate tissues from control and warfarin treated mice. We compared the transcriptomes of three control mice to four warfarin-treated mice, as one of the control-treated mouse samples did not meet quality control criteria. As expected, warfarin-treated and control datasets clustered together upon unsupervised hierarchical clustering (Figure 5A). We found that 732 genes were up-regulated and 676 genes were down-regulated by warfarin treatment. Among the most highly up and down-regulated genes (Figure 5B) are known androgen-regulated prostate genes, including secretoglobins (Scgb), which are involved in androgen binding [27], and seminal vesicle proteins (Svs), also important in prostate physiology [28, 29]. The AR transcript level was not found to be significantly altered by warfarin treatment. This suggests that warfarin is indeed regulating androgen signaling and AR activity. Interestingly, Spink3, the mouse homolog of Spink1 which is known to promote aggressive prostate cancer in humans, was also affected by wafarin [30].

Gene ontology analyses of significantly regulated genes suggested that warfarin inhibited AR activity, as both the AR and dihydrotestosterone signals were decreased by warfarin treatment. However, warfarin inhibited other pathways as well, some even more strongly than androgen signaling (Figure 5C–5E). Among the most significantly down-regulated pathways were those involved in lipid and cholesterol biosynthesis, as well as important regulators of these pathways including the peroxisome proliferator-activated receptor (PPAR) and sterol regulatory element-binding protein (SREBP) family [31]. The signal for the PPAR agonist troglitazone was also down-regulated by warfarin treatment. PPARs, in particular PPARγ, is deregulated in prostate cancer [32], and PPARγ may also regulate AR activity [33].

### PPAR mediates AR inhibition by warfarin

**Figure 6:**
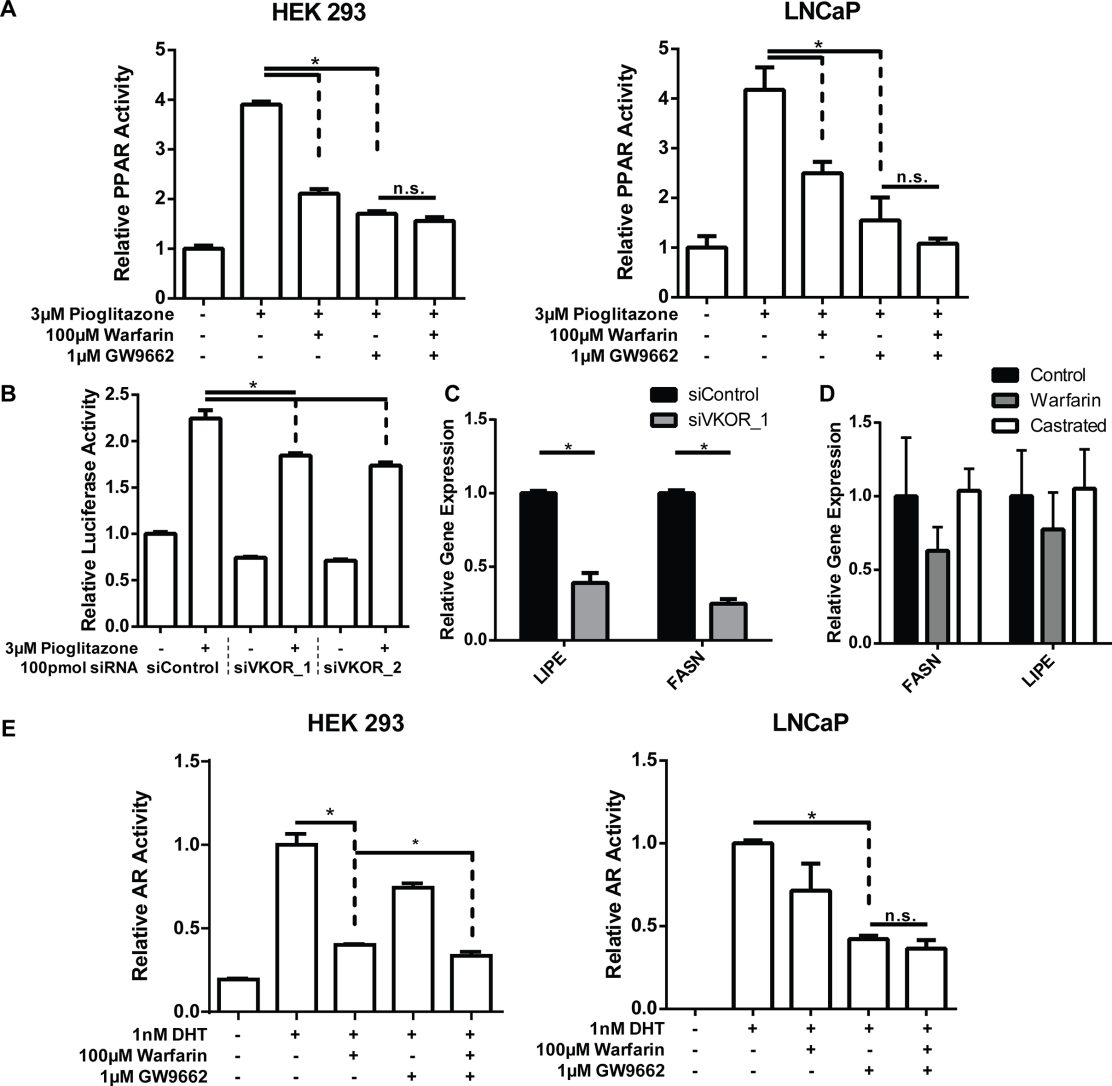
PPARγ inhibition is VKOR dependent and mediates AR inhibition (**A**) PPAR activity was measured in HEK293 CARY or LNCaP cells by luciferase reporter assay. Cells were transfected with PPAR sensitive PPRE-luciferase and renilla control reporters and treated with PPARγ selective agonist pioglitazone or antagonist GW9662. PPAR activity could no longer be inhibited by warfarin after GW9662 treatment. (**B, C**) VKOR was knocked down in HEK 293 cells and PPARγ activity was assessed by PPRE-luciferase activity (B) or the expression of PPARγ target genes (C). Decreased expression of VKOR inhibits PPARγ activity. (**D**) Expression of PPARγ target genes in mouse prostate measured by qPCR demonstrates a consistent but statistically insignificant trend toward inhibition with warfarin treatment that is not observed with castration. (**E**) AR activity was measured by PSA-luciferase in HEK 293 CARY and LNCaP cells as previously described. Simultaneous treatment of warfarin and GW9662 did not inhibit AR much further than the most effective single treatment.

To confirm that warfarin inhibits PPAR signaling, we first transfected cells with a firefly luciferase reporter driven by a PPAR response element (PPRE-luc) [34] as well as a control renilla luciferase reporter and treated HEK 293 and LNCaP cells with PPARγ selective agonists GW1929 or pioglitazone [35] and warfarin (Figure 6A). We found that, like with AR, warfarin inhibited PPARγ activity in a dose dependent manner in this assay, as did a known PPAR antagonist, GW9962 [36]. Knockdown of VKOR in HEK293 CARY cells also demonstrated that inhibition of PPARγ transcriptional activity and target gene expression is VKOR-dependent (Figure 6B, 6C). Like we observed with AR activity, VKOR knockdown inhibits both liganded (pioglitazone, pio) and unliganded PPARγ activity. RT-qPCR validation of our RNA-seq results also demonstrated that warfarin trends toward inhibition of PPARγ target genes in mice (Figure 6D). Importantly, castration (a positive control for inhibition of AR) did not affect the expression of these genes, suggesting that inhibition of AR is not upstream of inhibition of PPARγ. Because the PPARγ pathway has been shown to regulate the AR pathway, it is possible that inhibition of PPARγ signaling by warfarin is upstream of the effect on AR signaling. To test if PPARγ inhibition in turn inhibits AR activity, we transfected HEK 293 cells and LNCaP cells with PSA-luciferase and control plasmids and treated them with DHT and GW9962 (Figure 6E). We found that GW9962 did indeed inhibit AR activity, albeit with variable efficacy dependent on the cell line. Furthermore, we found that the combination of warfarin and GW9662 did not have an additive effect on the inhibition of AR activity, suggesting that both are in the same mechanistic pathway in regards to their effects on AR.

## DISCUSSION

Overwhelming evidence indicates that long term warfarin usage is associated with a decreased risk of PC. While this data is necessarily retrospective in nature as a prospective study testing the effects of the anticoagulant warfarin is not feasible due to safety concerns, the four largest experiments contain over 2.5 million subjects combined, and each reaches a very similar conclusion: warfarin usage of more than a half of a year significantly reduces the risk of a PC diagnosis. Although men on warfarin tend to be less healthy, and therefore less likely to undergo aggressive screening for PC, the association between warfarin use and decreased risk of PC is not likely attributable to ascertainment bias. Each study carefully controlled and adjusted for potential confounders and most importantly, the Schulman *et al*, study compared men with 6 months warfarin usage with 6 weeks of warfarin usage, thus mitigating any potential ascertainment bias [37]. Recall bias is also not an issue with these studies as prerecorded prescription histories were prospectively collected through patient registries. It is clear that warfarin is associated with decreased risk of PC.

Even though warfarin has not been studied prospectively in the context of prostate cancer prevention or treatment, coumarin, a related compound of the same class, has been shown to have some therapeutic properties against prostate cancer. Treatment of rats with high dose of coumarin was shown to significantly shrink the growth of prostate tumors, and at even caused the regression of the prostate, suggesting that coumarins such as warfarin could potentially have some antiandrogen properties [38]. When used at lower doses in patients however, its effect was less prominent and response was observed in only a few patients [39]. Here, we begin to unravel the mechanism by which warfarin works to prevent the development of prostate cancer.

Because of the primary role that vitamin K plays in the γ-carboxylation of proteins and our initial observation that warfarin can inhibit AR activity, we first determined if there were γ-carboxylation sites on AR. We utilized a previously described methodology specifically for identifying the location of γ-carboxylation on proteins [11]. Due to the instability of γ-carboxyglutamate and the interference of its highly negative charge with ionization, methylation was performed to both eliminate the negative charge and protect the carboxyl group from neutral loss. We were able to identify one γ-carboxylation site on residue E2 at the N-terminal domain of AR. The N-terminal domain contains the AF1 transactivation domain, which plays a role in the ligand-independent activation of AR [40] and is the location of a many post-translational modification sites. Our finding that mutation of the E2 γ-carboxylation site did not diminish the inhibition of AR activity by warfarin was unexpected, especially since E2 is known to regulate the activity of AR cofactor ART-27 [24]. In many γ-carboxylated proteins, γ-carboxylation sites usually occur in clusters, which helps in the binding of calcium ions to these proteins [25]. Not all glutamates were covered by our mass spectrometry analysis so we performed glutamate-scanning mutagenesis of many of these residues, including regions of where glutamates were clustered. We did not identify any glutamates that were important for warfarin-mediated AR inhibition, although we could not rule out this mechanism completely as not all glutamates were mutated singly or in combinations. Even though the γ-carboxylation of AR at E2 does not influence the transcriptional activity of AR under the conditions tested, it is likely that this post-translational modification has a role in AR regulation. As γ-carboxylation is known to coordinate calcium binding, which is important in bone homeostasis, γ-carboxylation of AR at E2 could contribute to the role AR signaling plays in bone [41]. Further studies would be required to elucidate the role of γ-carboxylation on AR. Importantly, AR is the first transcription factor found to undergo vitamin K-dependent γ-carboxylation.

Warfarin is the most widely used anticoagulant but dosing of warfarin is particularly difficult as the effective dose is highly variable between individuals [42] and warfarin users have to undergo frequent monitoring to ensure that the correct dose is given. Long-term use of warfarin also increases the risk of hemorrhages [43]. Because of these disadvantages, warfarin is unfit as a chemopreventive agent. However, we have demonstrated here that the warfarin-dependent γ-carboxylation of AR and its inhibition of AR transcriptional activity are unrelated, strongly suggesting that the anticoagulatory and chemopreventive effects of warfarin are separable. Mutation of the γ-carboxylation site of AR does not eliminate the effect of warfarin on AR, suggesting that the γ-carboxylation status of AR does not regulate its activity. This was further demonstrated by the knockdown of GGCX, which did not inhibit AR activity. Since the anticoagulatory activity of warfarin is driven by its inhibition of γ-carboxylation, the effects of warfarin on AR activity would be unrelated to its anticoagulatory effects. This remains to be definitely proven in animals models of prostate cancer. Interestingly, the knockdown of GGCX increased AR activity, again suggesting that γ-carboxylation of AR affects its activity in some way unrelated to warfarin-mediated inhibition of AR activity.

Because the anticoagulatory and chemopreventive effects of warfarin appear to be separable, it is likely possible to identify safer drug targets downstream of warfarin and develop novel chemopreventive agents against them. To identify potential candidates, we used RNA-seq to identify the signaling pathways affected by warfarin treatment in benign mouse prostate tissue. In addition to a downregulation of AR signaling, we observed very significant decreases in several pathways, many of which were related to PPAR signaling. Here we show that warfarin can inhibit PPAR signaling and that inhibition of PPARγ causes decreased AR activity. PPARγ is a well characterized regulator of AR activity, through its effects appear to be cell-type dependent [33], and exactly how it regulates AR activity is unclear [44]. One possible regulator is PPARγ coactivator 1-alpha (PGC-1α), a PPARγ-regulated transcriptional coactivator. PGC-1α was found to activate AR through interaction with the N-terminal domain of AR [45]. Although the authors did not further narrow down the region of interaction, it is possible that PGC-1α interaction could be mediated by γ-carboxylation, which remains to be determined.

Because warfarin has been documented to have chemopreventative properties distinct from treatment with 5α reductase inhibitors, namely decreased detection of high grade cancers in addition to low grade cancers, it could very well be that warfarin's effects on both PPARγ inhibition and other signaling pathways contributes to chemoprevention by mechanisms in addition to AR inhibition. Vitamin K signaling is known to regulate lipid biosynthesis through binding and activation of the steroid and xenobiotic receptor (SXR) [17]. Activation of SXR by vitamin K reduced steroid biosynthesis while promoting cholesterol efflux in the prostate. A similar phenotype was observed with the inhibition of PPARγ in castration-resistant prostate cancer cells [46]. It is therefore possible that inhibition of the vitamin K cycle by warfarin would reduce the steroidogenic potential of prostate through its effects on PPARγ. The upregulation of steroid biosynthesis is a known mechanism of castration resistance in prostate cancer. In the scope of prostate cancer initiation, upregulation of these pathways has been suggested to be a key step in increasing the sensitivity of the prostate to androgens and predispose it to tumorigenic changes [47]. These data suggest that PPARγ could be an important drug target for the chemoprevention of prostate cancer and warrant testing in prostate cancer mouse models to determine the effectiveness of targeting this pathway in a chemoprevention setting.

## MATERIALS AND METHODS

### Reagents

Dihydrotestosterone was purchased from Steraloids. Vitamin K antagonists were purchased from: warfarin sodium (TCI Chemicals), scopoletin (Sigma), brodifacoum (Sigma). Bicalutamide was purchased from Sigma. PPAR agonists and antagonists were purchased from: GW1929 (Sigma), Pioglitazone (Santa Cruz), GW9662 (Sigma).

### Cell lines and culture conditions

Human embryonic kidney cell line (HEK 293) and prostate adenocarcinoma cell lines (LNCaP) were purchased from ATCC. BPH-1 cells were a gift from Ann Donjacour. HEK 293 stably expressing fluorescent (CARY) and HA-tagged AR were generated previously [18]. Mouse prostate cancer cell line (E8) was generously provided by Dr. Roy Burman [21]. LNCaP cells were maintained in phenol red-free RPMI 1640 supplemented with 10% FBS and antibiotics. HEK293 and E8 cells were maintained in Dulbecco's modified Eagle's medium supplemented with 10% FBS and antibiotics.

### Transfection and transcriptional assays

Cells were transfected using Lipofectamine Plus (Thermofisher) with PSA-luciferase [48], MMTV-luciferase or PPRE-luciferase, and pRL-SV40 (Promega) as a control. Gene specific or negative control siRNA (Qiagen) was transfected together with the plasmids when applicable. Cells were transferred to a 96-well plate 24 hours after transfection and treated with the appropriate drugs dissolved in media supplemented with charcoal-stripped serum for another 24 hours. Fluorescence energy transfer (FRET) assay on CARY-expressing cells were performed as previously described[18]. Luciferase activity was assayed 24 hours after treatment using the dual-luciferase reporter assay system (Promega). Student's *t-test* (two-sided and equal variance) was performed and association was considered significant when *P* < 0.05 and indicated by an asterisk.

### Cell proliferation assays

For growth curves, LNCaP cells were transferred to charcoal stripped (C/S) media 3 days before they were split and plated at a density of approximately 20,000 cells/well in 48-well plates, in quadruplicate. The following day, medium with or without DHT and drugs was added to the cells. Proliferation was determined by measuring the DNA content of the cells in each well. Cells were fixed in 2% PFA, followed by staining for 5 min at room temperature with 0.2 ng/mL DAPI in PBS solution. The cells were washed with PBS solution, then read on a fluorescence plate reader using 365/439 excitation/emission wavelengths. A Student *t* test was used to determine significant differences between DHT treated and drug treated populations.

### Site-directed mutagenesis

Site-directed mutagenesis was performed using PCR with primers containing the desired mutations. AR expression plasmid was amplified with mutant primers using KAPA High Fidelity polymerase (Kapabiosystems) using manufacturer's protocol. Parent plasmid was digested with DpnI (Agilent) for 1 hour. Mutant plasmids were transformed into NEB 5-alpha competent cells (NEB). Mutations were screened by Sanger sequencing.

### Immunoprecipitation

LNCaP cells were treated with drugs for 24–72 hours and lysed in TBS, 0.1% Triton X-100, protease and phosphatase inhibitors (Roche). Immunoprecipitation was performed using anti-AR (AR441, Santa Cruz) or anti-GLA antibody (REF 3570, Sekisui Diagnostics). Western blot was used to detect AR (PG-21, Millipore), GLA or ART-27 (bs-6749R, Bioss).

### Warfarin treatment of nude mice

All animal experiments were conducted with approval from the institutional Animal Care and Use Committee of City of Hope. Warfarin treatment was performed by dissolving warfarin in drinking water at the desired concentrations. Male nude mice, aged 8 weeks, were obtained from the NCI breeding program. Mice were either treated with warfarin, surgically castrated or left untreated as controls. Mice were euthanized after 4 weeks and the prostate was harvested. Coagulation time was measured by sliding newly acquired blood in a glass capillary tube until it no longer slides or remained uncoagulated after 10 minutes.

### Quantitative PCR (qPCR)

Total RNA was extracted from cells or homogenized tissue using GeneJet RNA purification kit (ThermoFisher). Reverse transcription was performed using M-MLV reverse transcriptase (ThermoFisher). Gene expression was quantified using SYBR green (ThermoFisher) with Rox reference dye (ThermoFisher) on a StepOne Real Time PCR System (ThermoFisher). Relative gene expression was calculated by ΔΔCT. Student's *t-test* was performed and association was considered significant when *P* < 0.05 and indicated by an asterisk.

### RNA-seq

RNA sequencing was performed by the City of Hope Integrative Genomics core facility. cDNA synthesis and library preparation was performed using TruSeq RNA Library prep kit in accordance with the manufacturer supplied protocols. Libraries were sequenced on the Illumina Hiseq 2500 with single read 40 bp reads. The 40-bp long single-ended sequence reads were mapped to the human genome (hg19) using TopHat and the frequency of Refseq genes was counted with customized R scripts. The raw counts were then normalized using trimmed mean of M values (TMM) method and compared using Bioconductor package “edgeR”. The average coverage for each gene was calculated using the normalized read counts from “edgeR”. Differentially regulated genes were identified using one-way ANOVA with linear contrasts to calculate *p*-values, and genes were only considered if the false discovery rate (FDR) was < 0.25 and the absolute value of the fold change was > 1.5. Gene ontology analyses were performed using GSEA [49], DAVID [50] and Ingenuity Pathway Analysis (Qiagen).

### Immunohistochemistry

Immunohistochemistry was performed using standard protocols. Antigen retrieval was performed on paraffin-embedded sections using citrate-based antigen unmasking solution (Vector Labs, Burlingame, CA). Slides were blocked with 10% normal goal serum, and then stained with VKOR antibody developed by Berkner [51] (diluted 1:200 in TBST) or normal rabbit IgG (Santa Cruz) overnight at 4°C. Slides were then incubated in biotinylated anti-rabbit secondary antibody (Vector Labs) followed by Vectastain Elite ABC reagent (Vector Labs) and developed using DAB substrate (Vector Labs). Sections were counterstained with Harris hematoxylin (Poly Scientific, Bay Shore, NY). Human prostate tissue was obtained from a benign section of a man with localized prostate cancer and was obtained with approval from the City of Hope Institutional Review Board under protocol 11058. Mouse prostate tissue was obtained from the mouse experiment described above.

### Identification of γ-carboxylation site by mass spectrometry

Immunoprecipitated AR was separated by SDS-PAGE and stained with SimplyBlue (Themofisher). Gel bands were excised and destained in ammonium bicarbonate. After disulfide bond reduction with 10 mM tris(carboxyethyl)phosphine and thiol alkylation with 50 mM iodoacetamide, gel bands were incubated with trypsin/Lys-C or chymotrypsin (Promega) overnight. Peptides were extracted with 0.1% TFA/70% acetonitrile and lyophilized. Methyl esterification was performed by incubating in 2M methanolic HCl for 1 hour at 20°C to improve detection of γ-carboxyl groups [11]. Samples were lyophilized again and resuspended in 0.1% formic acid. Mass spectrometric analyses of the digest peptides were conducted on an Orbitrap Fusion hybrid mass spectrometer (Thermo Fisher) equipped with an Easynano UHPLC, using a 75 μm × 250 mm Pepmap RSLC reverse phase column with a PepMap 1000 trapping column (Thermo Fisher). 10 μl of methylated peptide samples were loaded at 4 μl per minute. LC was performed with a gradient mobile phase system containing buffer A (0.1% formic acid) and buffer B (100% acetonitrile/0.1% formic acid). A 40 minute gradient elution from the analytical column was conducted from 3% to 80% buffer B, followed by 45–60 minutes at 90% solvent B. Flow rate was 300 nl/min. Full mass scans (200–4000 Da) were taken by the Orbitrap mass analyzer, operated at 120K resolution, while collision-induced dissociation (CID) was conducted in data-dependent mode to generate MS/MS data. The data was analyzed using PEAKS Studio (Bioinformatics Solutions Inc.) and Proteome Discoverer (Thermo Fisher) using a non-redundant human protein database (Swissprot and NR) with the tagged AR sequence added. Database searches were carried out by considering three missed enzymatic cleavages, a precursor ion mass tolerance of 5 ppm and 0.02 Da mass tolerance for fragment ions. Search parameters also included cysteine carbamidomethylation, methionine oxidation, glutamate carboxylation, and carboxy methyl esters were searched as expected amino acid modifications.

## SUPPLEMENTARY FIGURES


