# Supplementary material for: Vitamin K epoxide reductase regulation of androgen receptor activity
Source: Oncotarget. 2017 Jan 13;8(8):13818–31. doi: 10.18632/oncotarget.14639 (PMC5355141; doi:10.18632/oncotarget.14639)
Supplement: Supplementary file 1 [file oncotarget-08-13818-s001.pdf]

Vitamin K epoxide reductase regulation of androgen receptor activity

Supplementary Materials

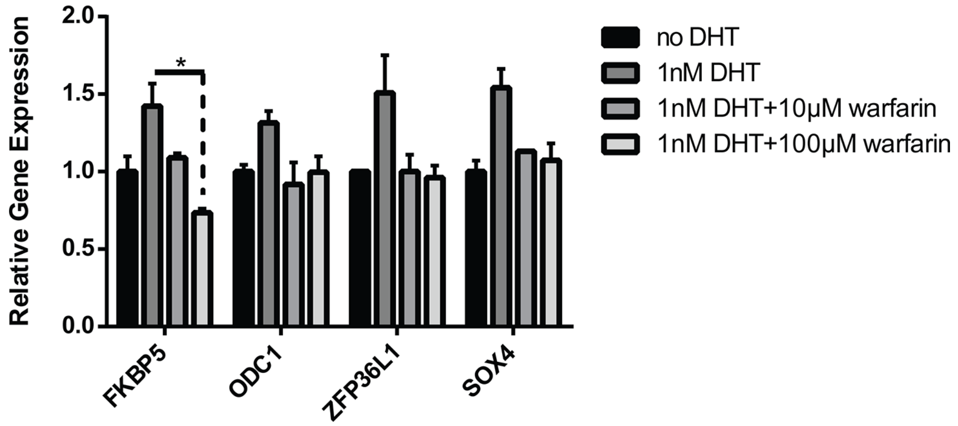

Supplementary Figure 1: HEK293 CARY cells were treated with the indicated drugs overnight. RNA was harvested and expression of known AR target genes was measured by RT-qPCR. Warfarin reduced the levels of the AR target genes.

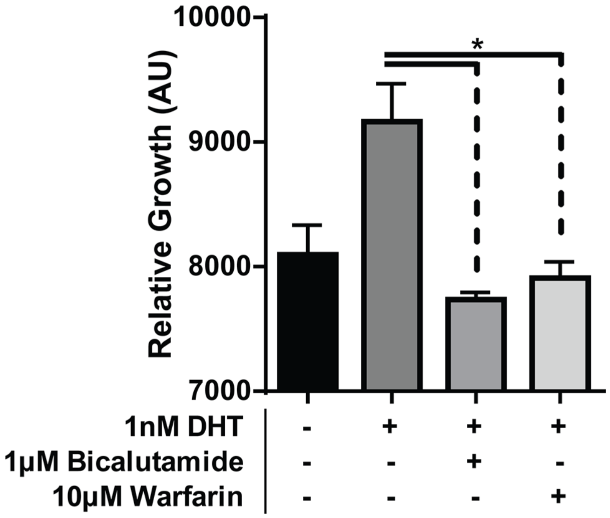

Supplementary Figure 2: LNCaP cells were plated in charcoal stripped media containing the indicated drugs in 48 well plates in quadruplicate. After seven days, cells were fixed, stained with DAPI, and DAPI content quantified on a fluorescent plate reader.

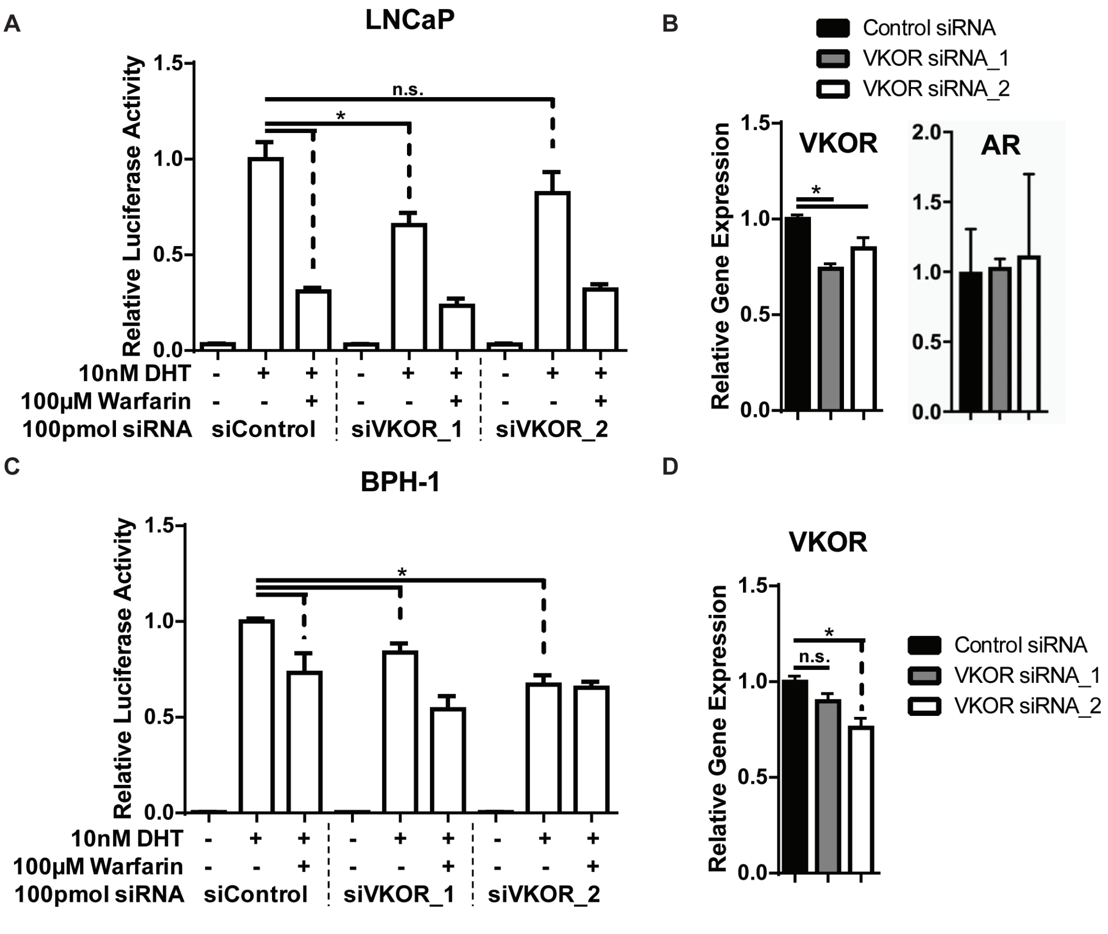

Supplementary Figure 3: VKOR was knocked down in LNCaP (**A**–**B**) and BPH-1 (**C**–**D**) cells using two different siRNAs. (A, C) Cells were transfected with siRNAs along with luciferase reporters. The following day, cells were treated with the indicated drugs. VKOR knockdown reduces AR transcriptional activity measured by the luciferase reporter assay. (B, D) The efficiency of the knockdown was measured by qPCR.

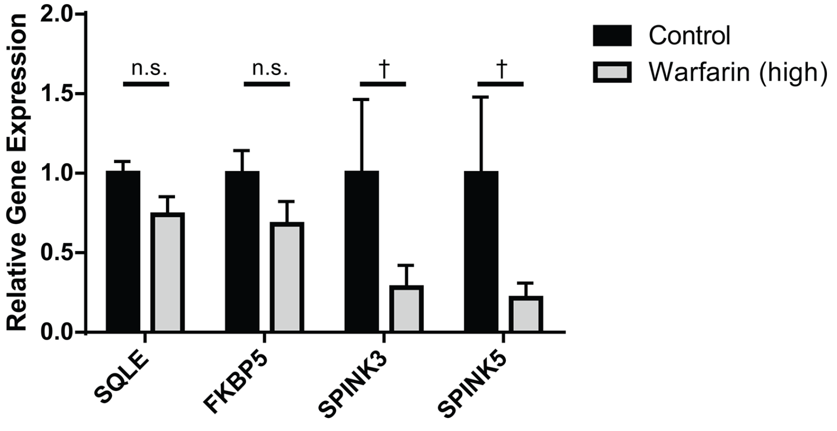

Supplementary Figure 4: Nude mice were treated with high (4.5 mg/L, *n* = 4) doses of warfarin in their drinking water or left intact (*n* = 9) as controls. After 4 weeks, the prostates were also harvested for RNA. The transcript levels of AR target genes were measured by RT-qPCR. Statistical significance was denoted by an asterisk (*) for $P < 0.05$ or a dagger (†) for $P < 0.1$.

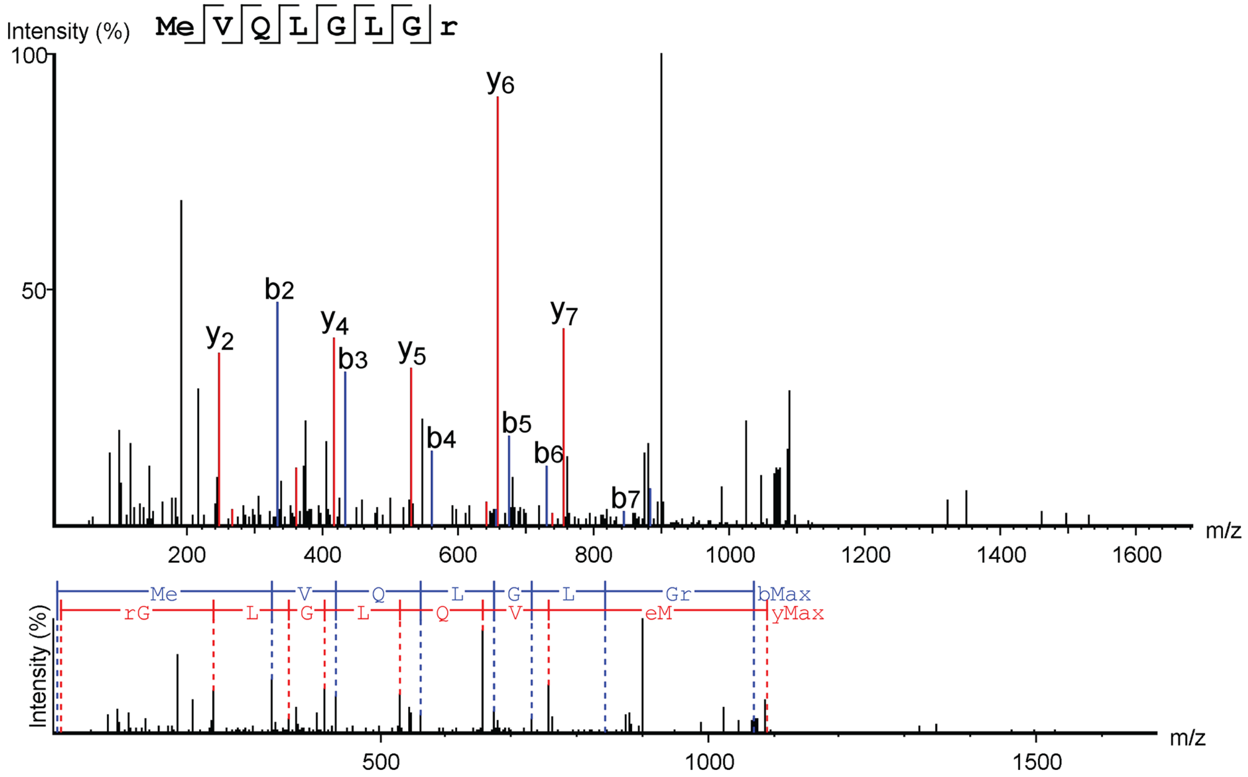

Supplementary Figure 5: Collision-induced dissociation (CID) spectra showing site of AR γ-carboxylation. Collision-induced dissociation (CID) spectra of peptide containing residues 1–9 revealed a Gla residue represented by the letter "e", that is both γ-carboxylated and has two additional methyl groups.

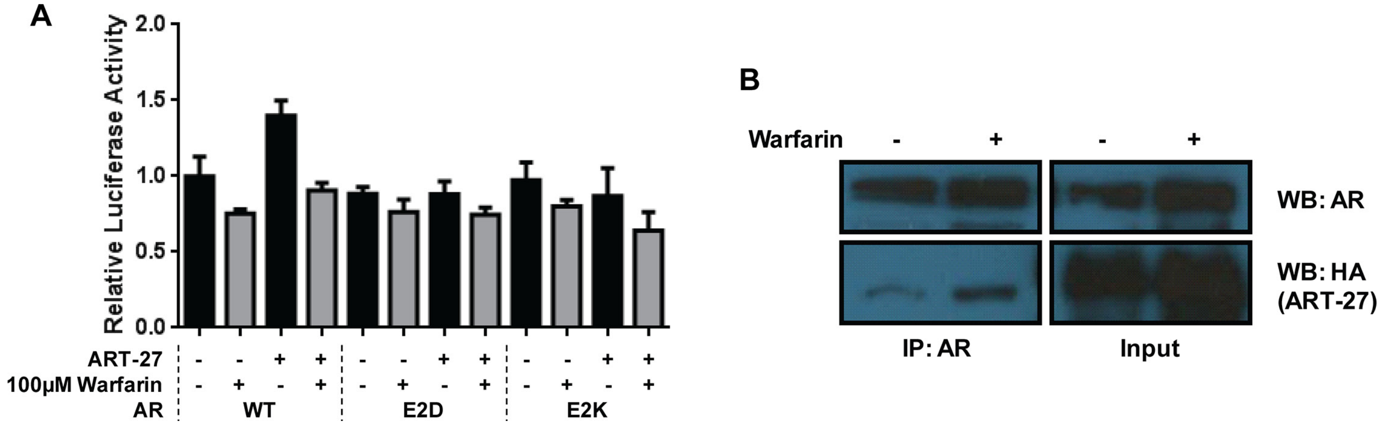

Supplementary Figure 6: Warfarin does not mediate the activation of AR by ART-27. (**A**) HEK293 cells transfected with wild type or E2 mutant AR and ART-27 were assayed for AR activity as previously described. AR E2 mutants were insensitive to ART-27 overexpression, but warfarin was capable of inhibiting wild-type AR activation by ART-27. (**B**) HEK 293 cell expressing CFP- and YFP-tagged AR and HA-tagged ART-27 were lysed and co-immunoprecipitation was performed on AR. HA-ART27 was detected by western blot.
